# Associations between polyunsaturated fatty acid concentrations and Parkinson’s disease: A two-sample Mendelian randomization study

**DOI:** 10.3389/fnagi.2023.1123239

**Published:** 2023-02-22

**Authors:** Xue Zhu, Sijia Huang, Wenyan Kang, Peizhan Chen, Jun Liu

**Affiliations:** ^1^Department of Neurology and Institute of Neurology, Ruijin Hospital, Shanghai Jiao Tong University School of Medicine, Shanghai, China; ^2^Department of General Surgery, Ruijin Hospital, Shanghai Jiao Tong University School of Medicine, Shanghai, China; ^3^Co-innovation Center of Neuroregeneration, Nantong University, Nantong, China

**Keywords:** polyunsaturated fatty acids, Parkinson’s disease, Mendelian randomization, causality, dietary modification

## Abstract

**Introduction:**

Observational studies demonstrated controversial effect of polyunsaturated fatty acids (PUFAs) on Parkinson’s disease (PD) with limited causality evidence. Randomized control trials showed possible improvement in PD symptoms with PUFA supplement but had small study population and limited intervention time.

**Methods:**

A two-sample Mendelian randomization was designed to evaluate the causal relevance between PUFAs and PD, using genetic variants of PUFAs as instrumental variables and PD data from the largest genome-wide association study as outcome. Inverse variance weighted (IVW) method was applied to obtain the primary outcome. Mendelian randomization Egger regression, weighted median and weighted mode methods were exploited to assist result analyses. Strict Mendelian randomization and multivariable Mendelian randomization (MVMR) were used to estimate direct effects of PUFAs on PD, eliminating pleiotropic effect. Debiased inverse variance weighted estimator was implemented when weak instrument bias was introduced into the analysis. A variety of sensitivity analyses were utilized to assess validity of the results.

**Results:**

Our study included 33,674 PD cases and 449,056 controls. Higher plasma level of arachidonic acid (AA) was associated with a 3% increase of PD risk per 1-standard deviation (SD) increase of AA (IVW; Odds ratio (OR)=1.03 [95% confidence interval (CI) 1.01-1.04], *P* = 2.24E-04). After MVMR (IVW; OR=1.03 [95% CI 1.02-1.04], *P* =6.15E-08) and deletion of pleiotropic single-nucleotide polymorphisms overlapping with other lipids (IVW; OR=1.03 [95% CI 1.01-1.05], *P* =5.88E-04), result was still significant. Increased level of eicosapentaenoic acid (EPA) showed possible relevance with increased PD risk after adjustment of pleiotropy (MVMR; OR=1.05 [95% CI 1.01-1.08], *P* =5.40E-03). Linoleic acid (LA), docosahexaenoic acid (DHA), docosapentaenoic acid (DPA) and alpha-linolenic acid (ALA) were found not causally relevant to PD risk. Various sensitivity analyses verified the validity of our results. In conclusion, our findings from Mendelian randomization suggested that elevated levels of AA and possibly EPA might be linked to a higher risk of PD. No association between PD risk and LA, DHA, DPA, or ALA was found.

**Discussion:**

The odds ratio for plasma AA and PD risk was weak. It is important to approach our results with caution in clinical practice and to conduct additional studies on the relationship between PUFAs and PD risk.

## 1. Introduction

Parkinson’s disease (PD) is one of the most common neurodegenerative diseases in the world. Disease-modifying treatment, such as Mediterranean diet and other nutritional strategies, were tempted on PD patients but still lack robust evidence (Alcalay et al., 2012; GBD 2016 Neurology collaborators, 2019; Metcalfe-Roach et al., 2021). Polyunsaturated fatty acids (PUFAs) were one of the primarily focused disease-modifying factors in the dietary treatment. PUFAs are believed to regulate the structure and function of neurons and glial cells (Bazinet and Layé, 2014; Fu et al., 2022), thus may help preserve the significantly lost substantia nigra cells in PD patients. Altered dietary intake of PUFAs and altered PUFA metabolism were reported in a range of neurological and psychiatric disorders, including Parkinson’s disease (Bazinet and Layé, 2014; Yoo et al., 2021). Linoleic acid (LA) and alpha-linolenic acid (ALA), two common PUFAs, are obtained through the diet and act as precursors. Through elongation and desaturation, LA is converted into arachidonic acid (AA), termed omega-6 PUFA. ALA is converted into eicosapentaenoic acid (EPA), docosapentaenoic acid (DPA), and docosahexaenoic acid (DHA), termed omega-3 PUFAs. The two predominant PUFAs in the brain are omega-6 AA and omega-3 DHA (Bazinet and Layé, 2014).

The effect of PUFAs on PD risk was controversial among studies. Intake of omega-3 PUFAs or ALA was inversely associated with PD risk in one case–control study, but demonstrated no association with PD when adapted to another population (de Lau et al., 2005; Dong et al., 2014; Kamel et al., 2014). Higher intake of AA demonstrated increased risk of PD in one case–control study, but not in the others (Miyake et al., 2010; Kamel et al., 2014). The above four small-sample case–control studies were highly subjective to selection bias, confounding and reverse causality, while prospective population-based cohort study, with larger sample sizes and longer observational time, may exhibit more reliable causality. Intake of total PUFAs, ω-3 PUFAs, or ALA was found to be associated with a lower risk of PD in one cohort (de Lau et al., 2005), and a weak positive association was shown between omega-6 PUFA intake and the risk of PD in a similar cohort (Dong et al., 2014). In a third cohort with 15-year observation, no statistically significant association was shown between dietary omega-3 and omega-6 PUFA and PD risk (Tan et al., 2016). Apart from these inconsistent results, reverse causality due to poor dietary intake from illness or confounding factors due to social-economic status was still unable to be addressed in population-based prospective cohort. Although randomized control trials (RCTs) offer greater evidence in causality, RCTs about PUFAs and PD risk were still inconclusive. Improvement in PD symptoms was observed in patients with supplementation of mixed PUFAs, but these studies had limited populations (20–30 patients per group) and short observational time (3–30 months), thus the long-term effect of PUFAs and generalizability of the result was undetermined (Taghizadeh et al., 2017; Pantzaris et al., 2021). Moreover, RCTs are costly, time-consuming, and sometimes impractical to carry out (Emdin et al., 2017).

Accounting for the conundrum of paradoxical evidence in observational studies above and lack of robust RCTs, the causal relevance between PUFAs and PD risk was still vague. Mendelian randomization is a method that uses genetic variations as instrumental variables to strengthen the causal inference regarding exposures and outcomes. Instrumental variables are variables associated with exposures that are not related to confounders, and affect the outcome only through the exposures. Mendelian randomization studies are less susceptible to confounding and reverse causality bias than traditional observational epidemiology. By using summary-level statistics of each genetic variant from two non-overlapping databases, a two-sample Mendelian randomization provided better practicality than RCTs (Skrivankova et al., 2021). Therefore, we adopted this methodology to answer the question about whether PUFAs were causally associated with PD risk.

## 2. Materials and methods

A valid Mendelian randomization (MR) analysis has three core assumptions: (1) The variant is associated with the exposure. (2) The variant is not associated with any confounder of the exposure-outcome association. (3) The variant does not affect the outcome, except *via* its association with the exposure. A fourth additional homogeneity assumption is required for estimating effect sizes through instrumental variable approaches: (4) There is a constant causal effect of the exposure of interest on the outcome across population (Skrivankova et al., 2021).

### 2.1. PUFA exposure data

Instruments for relative blood concentrations (percentage of total fatty acids) of six PUFAs were selected from 19 cohorts of three genome-wide association studies (GWAS) totaling 22,393 individuals (Lemaitre et al., 2011; Guan et al., 2014; Kettunen et al., 2016). Details of the contributing researches are summarized in Supplementary Tables S1, S2. All participates were of European ancestry.

The exclusion criteria for single-nucleotide polymorphisms (SNPs) included failing to reach the significant genome-wide association level 5E-08 and showing a minor allele frequency <0.01. After harmonization of instrument SNPs with corresponding outcome data as well as linkage disequilibrium clumping (*r*^2^ = 0.001, strand alignment = 10,000 kb), there remained 31 independent SNPs for AA, 54 SNPs for LA, 6 SNPs for DHA, 4 SNPs for EPA, 3 SNPs for DPA, and 1 SNP for ALA (Supplementary Table S3) as instrumental variables. If instrument SNPs were not found in the outcome data, proxy variants (*r*^2^ > 0.8) were exploited using the online webtool SNiPA^1^ (Coneys et al., 2021).

### 2.2. PD outcome data

We acquired summary statistics from the latest and largest GWAS meta-analysis for PD risk (33,674 cases and 449,056 controls of European ancestry; Nalls et al., 2019) and no participants overlap existed between exposure and outcome database. Detailed information of the PD outcome GWAS meta-analysis was delineated in the appendix (Supplementary Tables S1, S4). Ethical approvals and written informed consent of all the participants involved in our study were stated in the original publications.

### 2.3. Mendelian randomization analyses

Four Mendelian randomization analyses were adopted: inverse variance weighted (IVW) method, Mendelian randomization Egger regression (MR-Egger) method, weighted median method, and weighted mode method. If all genetic variants satisfy the instrumental variable assumptions, then IVW method is an appropriate estimate of the causal effect and is used as the primary analysis (Burgess and Thompson, 2011). Under InSIDE assumption that requires instrument strength being independent of direct effect, MR-Egger method serves as a powerful tool when all the genetic variants are invalid instrumental variables, and the nonzero intercept can indicate a net horizontal pleiotropic effect (Bowden et al., 2015). Weighted median method assumes that no single instrumental variable contributes more than 50% of the weight, so the analysis is credible when 50% of weight contributed by genetic variants is valid (Bowden et al., 2016a). Weighted mode method supposes that the largest number of similar individual-instrument causal effect estimates come from valid instruments. It is reliable even if the majority of instruments are invalid (Hartwig et al., 2017). We also searched online webtool Phenoscanner^2^ to determine pleiotropic SNPs which were also significantly (*p =* 5E-08) associated with other lipids (Supplementary Table S5; Yavorska and Burgess, 2017). Then, multivariable Mendelian randomization (MVMR) method was utilized to eliminate bias from these confounding factors. As this method requires more than three SNPs to analyze, MVMR was not applied for DPA and ALA (Sanderson et al., 2021). In order to avoid potential bias to the utmost extent, pleiotropic SNPs selected from Phenoscanner were deleted from instrument sets of AA and LA before analyzing, and this method was called strict Mendelian randomization (Holmes et al., 2015). Omega-3 PUFAs had limited SNPs and subtraction of SNPs were not appropriate, so MVMR was not implemented. To include larger instrument sets for omega-3 PUFAs and make result comparison, we released the selection threshold of *p*-value up to 5E-06. Weak instrument bias was introduced through this practice, so debiased inverse variance weighted (IVW) estimator was implemented to evaluate the validity of the results (Ye et al., 2021). False discovery rate (FDR) correction was applied to provide justification (Sesia et al., 2021).

IVW, MR-Egger, and MVMR methods all demand NO Measurement Error (NOME) assumption, where SNP-exposure associations are already known rather than estimated. F-statistic was calculated using the following formula (Papadimitriou et al., 2020):


$$F=\frac{R^{2}\left(N-2\right)}{1-R^{2}}$$

Here, *R*^2^ is the variance of the risk of PD explained by each instrument and N is the sample size of PUFA GWAS database. *R*^2^ was calculated as follows (Papadimitriou et al., 2020):


$$R^{2}=\frac{2\times \mathrm{EAF}\times \left(1-\mathrm{EAF}\right)\times {\mathrm{beta}}^{2}}{2\times \mathrm{EAF}\times \left(1-\mathrm{EAF}\right)\times {\mathrm{beta}}^{2}+2\times \mathrm{EAF}\times \left(1-\mathrm{EAF}\right)\times N\times \mathrm{SE}{\left(\mathrm{beta}\right)}^{2}}$$

Here, EAF is the effect allele frequency, beta is the estimated genetic effect on PUFA, and SE (beta) is the standard error of the genetic effect. Mean F-statistics is widely used to measure the weak instrument bias whenever the genetic variants violate the NOME assumption. Generally, mean F-statistics is required to be more than 10 to avoid weak instrument bias (Burgess and Thompson, 2011). As for MR-Egger regression, *I*^2^_GX_ was used to assess the effect of NOME violation. When *I*^2^_GX_ is <90%, causal effect estimated from MR-Egger method should be interpreted with caution due to regression dilution. Bias adjustment is done *via* simulation extrapolation (SIMEX) method under such condition (Küchenhoff et al., 2006; Bowden et al., 2016b). We also used conditional F-statistics to test the strength of the instruments in MVMR analysis (Sanderson et al., 2021). Statistical power was calculated using the mRnd power calculation tool, assuming the true causal odds ratio (OR) was 1.05 (Brion et al., 2013).

The Mendelian Randomization Pleiotropy RESidual Sum and Outlier (MR-PRESSO) method was utilized to search for pleiotropic outlier SNPs, and pooled estimates were adjusted if necessary (Verbanck et al., 2018). rs780094 was deleted as an outlier for DPA and left only 2 SNPs as instrumental variables. Mendelian randomization pleiotropy test was employed to assess horizontal pleiotropy. Presence of heterogeneity between individual SNP effect estimate was evaluated using Cochrane’s *Q* test and *I*^2^ for IVW analysis (0–25% *I*^2^ means no heterogeneity; 25–50% *I*^2^ means low heterogeneity; 50–75% *I*^2^ means moderate heterogeneity; 75–100% *I*^2^ means substantial heterogeneity), and a fixed-effects model (IVW) was applied if the heterogeneity was low to moderate (*I*^2^ < 50%); otherwise, a random-effects model was used (*I*^2^ ≥ 50%; Higgins and Thompson, 2002; Greco et al., 2015). Scatter plots depicted the association of the exposure effect versus the outcome effect, and the slope of the line corresponded to the estimated causal effect. Forest plots exhibited the causal estimate obtained from each genetic variant, allowing for a direct visualization of heterogeneity in the results. Leave-one-out plots performed multiple times of Mendelian randomization by excluding one SNP at each analysis and were commonly used in heterogeneity analysis. In funnel plots, causal effect for genetic variants were plotted against precisions, and asymmetry indicated horizontal pleiotropy (Skrivankova et al., 2021).

### 2.4. Statistical analyses

All statistical analyses were performed using R software (version 4.1.2). IVW, MR-Egger, weighted median, and weighted mode analyses were performed using the R package TwoSampleMR (version 0.5.6; Hemani et al., 2018a), debiased IVW was performed through R package MR.DIVW (Version 0.1.0; Ye et al., 2021), multivariable Mendelian randomization was performed through R package MVMR (Version 0.3.0; Sanderson et al., 2021), SIMEX bias adjustment was performed *via* R package SIMEX (Version 1.8; Küchenhoff et al., 2006), and pleiotropic outlier SNPs were selected *via* R package MRPRESSO (Version 0.1.0; Verbanck et al., 2018).

## 3. Results

We acquired exposure summary-level data from 22,393 participants and outcome data from 33,674 cases and 449,056 controls (Supplementary Tables S2, S4). All participants were of European ancestry and no participants overlap existed between exposure and outcome data.

Mean F-statistics of all PUFAs ranged from 51 to 401, indicating that IVW estimates were not violated by weak instrument bias. Conditional F-statistics showed that MVMR of AA, LA, DHA, and EPA (conditional *F* = 90, 35, 46, 10) also did not succumb to weak instrument bias. The total variance explained by each instrumental exposure ranged from 2.81 to 38.34%. Omega-6 PUFAs (38.34% for AA; 31.10% for LA) showed better robustness in the association between genetic variants and exposures than omega-3 PUFAs. Power calculation also indicated higher reliability of AA and LA (1 for AA and LA). *I*^2^_GX_ of all PUFAs were more than 0.9, indicating no NOME violation in MR-Egger analysis, except for DHA (*I*^2^_GX_ = 0.67). So SIMEX adjustment was adopted on MR-Egger results of DHA, and the results were consistent (Table 1).

**Table 1:** Results of instrument strength assessment and debiased IVW.

PUFA	*N*	Mean F	Variance explained	Power	*I* ^2^_GX_	MR-Egger + SIMEX	Conditional F	Debiased IVW
AA	31	116	38.34%	1	0.99	–	90	–
LA	54	80	31.10%	1	0.99	–	35	–
DHA	6	51	3.42%	0.35	0.67	0.80 (−1.15–2.75), *p =* 0.85	46	0.97 (0.83–1.11), *p =* 0.69
EPA	4	94	4.14%	0.49	0.99	–	10	0.97 (0.87–1.08), *p =* 0.63
DPA	2	401	8.55%	0.76	0.99	–	–	1.00 (0.67–1.32), *p =* 0.98
ALA	1	256	2.81%	0.33	–	–	–	1.02 (0.33–1.72), *p =* 0.95

IVW, inverse variance weighted; PUFA, polyunsaturated fatty acids; N, number; MR, Mendelian randomization; SIMEX, simulation extrapolation; AA, arachidonic acid; LA, linoleic acid; DHA, docosahexaenoic acid; EPA, eicosapentaenoic acid; DPA, docosapentaenoic acid; ALA, alpha-linolenic acid.

**Figure 1:**
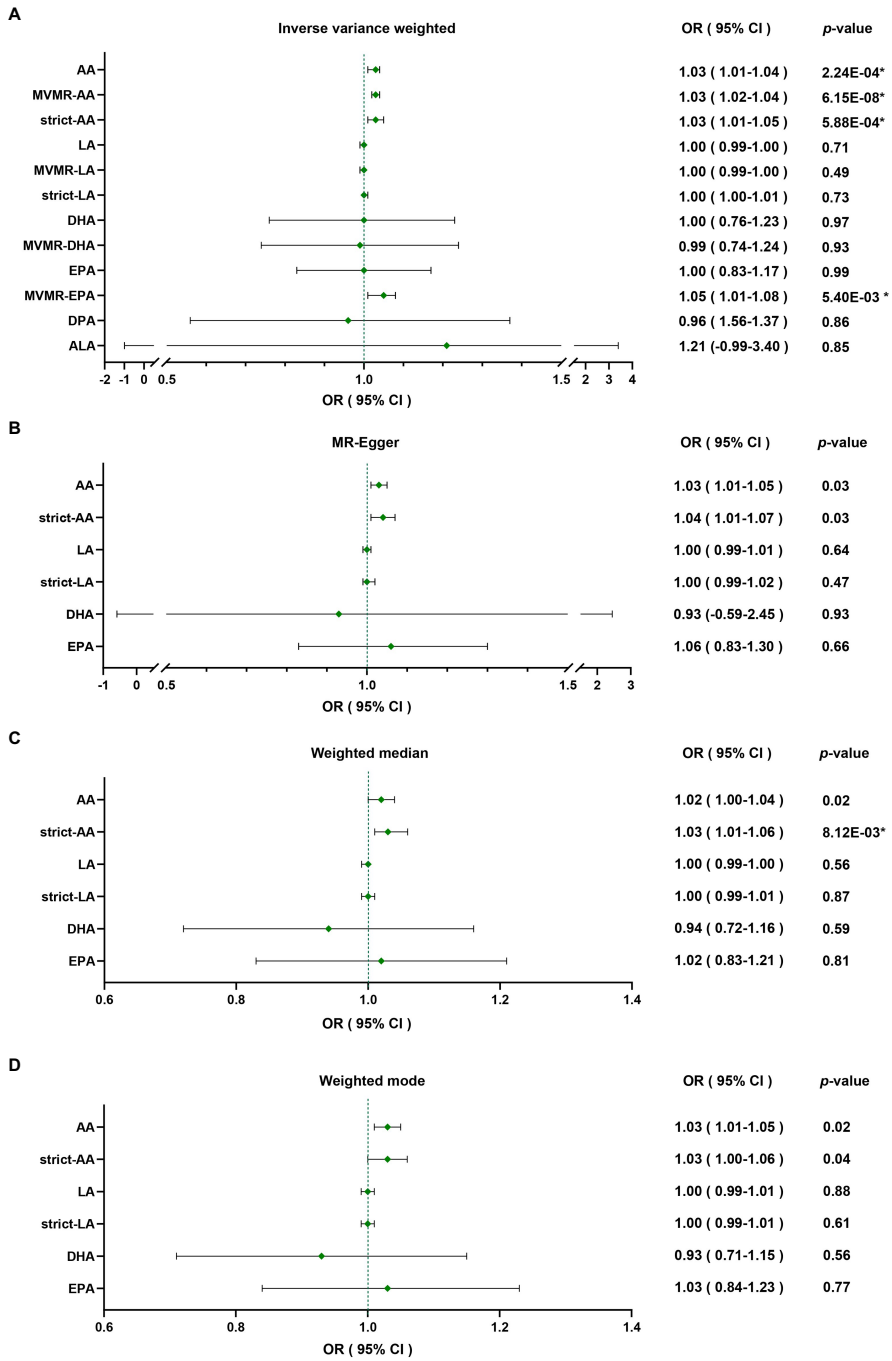
Forest map of inverse variance weighted, MR-egger, weighted median, weighted mode analyses: causal relevance between PUFAs and PD were analyzed through **(A)** Inverse variance weighted, **(B)** MR-Egger, **(C)** weighted median, **(D)** weighted mode. *Results with a significant *p-*value after false discovery rate correction. MR-Egger, Mendelian randomization Egger regression; MVMR, multivariable Mendelian randomization; OR, odds ratio; CI, confidence interval; AA, arachidonic acid; LA, linoleic acid; DHA, docosahexaenoic acid; EPA, eicosapentaenoic acid; DPA, docosapentaenoic acid; ALA, alpha-linolenic acid.

Through four common Mendelian randomization analyses, we found that genetically predicted high plasma level of AA was associated with a 3% increase of PD risk per 1-standard deviation (SD) increase of AA by IVW (OR = 1.03 [95% confidence interval (CI) 1.01–1.04], *p =* 2.24E-04; Table 2). As some of the SNPs were also significantly associated with other lipids through Phenoscanner searching (Supplementary Table S5), which could also interrelate with PD risk, we applied MVMR to reduce the pleiotropic effect; furthermore, deletion of these pleiotropic SNPs was also implemented to minimize the potential bias. Results of MVMR (IVW; OR = 1.03 [95% CI 1.02–1.04], *p =* 6.15E-08) and strict Mendelian randomization (IVW; OR = 1.03 [95% CI 1.01–1.05], *p =* 5.88E-04) were both consistent with primary analysis. LA demonstrated no causal relevance with PD risk (IVW; OR = 1.00 [95% CI 0.99–1.00], *p =* 0.71), and results were consistent after MVMR (IVW; OR = 1.00 [95% CI 0.99–1.00], *p =* 0.49) and strict Mendelian randomization (IVW; OR = 1.00 [95% CI 1.00–1.01], *p =* 0.73). Omega-3 PUFAs were also not causally linked with PD (IVW; OR = 1.00 [95% CI 0.76–1.23], *p =* 0.97 for DHA; OR = 1.00 [95% CI 0.83–1.17], *p =* 0.99 for EPA; OR = 0.96 [95% CI 0.56–1.37], *p =* 0.86 for DPA; and OR = 1.21 [95% CI-0.99-3.40], *p =* 0.85 for ALA). Then, MVMR was applied for DHA and EPA. DHA MVMR results were consistent with univariable Mendelian randomization, but EPA was shown to be a risk factor for PD after the adjustment (IVW; OR = 1.05 [95% CI 1.01–1.08], *p =* 5.40E-03; Table 2), which indicated EPA as a possible PD risk factor. Given that instrument sets of omega-3 PUFAs were small, debiased IVW was implemented after including independent SNPs meeting *p*-value of 5E-06 or less into the instrument sets, and the results were in accordance with classical IVW Mendelian randomization analyses (Table 1). Results were plotted in the forest map (Figure 1). Scatter plots were exhibited in the supplementary for direct visualization of the results (Supplementary Figures S1–S5).

**Table 2:** MR analyses between exposures (PUFAs) and outcome (PD Risk).

	Exposure	*N*	Inverse variance weighted			MR-Egger		
			OR	95%CI	Value of *p*	OR	95%CI	Value of *p*
omega-6 PUFAs	AA	31	1.03	1.01–1.04	2.24E-04^*^	1.03	1.01–1.05	0.03
	MVMR-AA	31	1.03	1.02–1.04	6.15E-08^*^			
	strict-AA	19	1.03	1.01–1.05	5.88E-04^*^	1.04	1.01–1.07	0.03
	LA	54	1.00	0.99–1.00	0.71	1.00	0.99–1.01	0.64
	MVMR-LA	54	1.00	0.99–1.00	0.49			
	strict-LA	37	1.00	1.00–1.01	0.73	1.00	0.99–1.02	0.47
omega-3 PUFAs	DHA	6	1.00	0.76–1.23	0.97	0.93	−0.59 to 2.45	0.93
	MVMR-DHA	6	0.99	0.74–1.24	0.93			
	EPA	4	1.00	0.83–1.17	0.99	1.06	0.83–1.30	0.66
	MVMR-EPA	4	1.05	1.01–1.08	5.40E-03^*^			
	DPA	2	0.96	0.56–1.37	0.86	–		
	ALA	1	1.21	−0.99 to 3.40	0.85	–		
	**Exposure**	***N***	**Weighted median**			**Weighted mode**		
			**OR**	**95%CI**	**Value of *p***	**OR**	**95%CI**	**Value of *p***
omega-6 PUFA	AA	31	1.02	1.00–1.04	0.02	1.03	1.01–1.05	0.02
	strict-AA	19	1.03	1.01–1.06	8.12E-03^*^	1.03	1.00–1.06	0.04
	LA	54	1.00	0.99–1.00	0.56	1.00	0.99–1.01	0.88
	strict-LA	37	1.00	0.99–1.01	0.87	1.00	0.99–1.01	0.61
omega-3 PUFA	DHA	6	0.94	0.72–1.16	0.59	0.93	0.71–1.15	0.56
	EPA	4	1.02	0.83–1.21	0.81	1.03	0.84–1.23	0.77
	DPA	2			–	–		–
	ALA	1			–	–		–

MR, Mendelian randomization; PUFA, polyunsaturated fatty acid; PD, Parkinson’s disease; MVMR, multivariable Mendelian randomization; MR-Egger, Mendelian randomization Egger regression; N, number of SNPs included; OR, odds ratio; CI, confidence interval; AA, arachidonic acid; LA, linoleic acid; DHA, docosahexaenoic acid; EPA, eicosapentaenoic acid; DPA, docosapentaenoic acid; ALA, alpha-linolenic acid. *Results with a significant *p*-value after false discovery rate correction.

With regard to sensitivity analyses, there was no potential pleiotropic outlier SNP observed in PUFA-PD association using MR-PRESSO (*p* ranging from 0.1 to 0.99). Pleiotropy was not detected in all PUFAs *via* pleiotropic test (*p* ranging from 0.42 to 0.97). Funnel plots of PUFAs (AA, LA) were generally symmetrical, which was another indication of no horizontal pleiotropy (Supplementary Figures S1, S2). Heterogeneity was assessed *via* Q test, and only DHA was found to be moderately heterogeneous (*p* for Cochrane’s *Q* = 0.05; *I*^2^ = 54%). *I*^2^ equaling to about 50% represented that half of the variation was due to between-study heterogeneity (von Hippel, 2015), and this was also shown in leave-one-out plot (Table 3; Supplementary Figure S3). Additionally, forest plots and leave-one-out plots for other PUFAs indicated no heterogeneity (Supplementary Figures S1, S2, S4, S5).

**Table 3:** Results of sensitivity analyses.

	*N*	*p-*value for MR-PRESSO	*p-*value for pleiotropy test	*p-*value for Cochran’s Q	*I* ^2^
AA	31	0.99	0.97	0.99	0%
LA	54	0.47	0.42	0.45	0%
DHA	6	0.10	0.94	0.05	54%
EPA	4	0.86	0.54	0.86	0%
DPA	2	–	–	0.95	0%
ALA	1	–	–	–	–

N, number of SNPs included; MR-PRESSO, Mendelian Randomization Pleiotropy RESidual Sum and Outlier; AA, arachidonic acid; LA, linoleic acid; DHA, docosahexaenoic acid; EPA, eicosapentaenoic acid; DPA, docosapentaenoic acid; ALA, alpha-linolenic acid.

## 4. Discussion

Mendelian randomization possesses unique characteristics including strong causal relevance, few confounding factors, no reverse causality, low expenses, and limited ethical restrictions (Skrivankova et al., 2021). Moreover, the evidence strength hierarchy of Mendelian randomization studies sits at the interface of observational and experimental studies (Davies et al., 2018). Nowadays, many observational studies and small-sample-size RCTs revealed controversial relationships between PUFAs and PD risk (de Lau et al., 2005; Miyake et al., 2010; Dong et al., 2014; Kamel et al., 2014; Tan et al., 2016; Taghizadeh et al., 2017; Pantzaris et al., 2021), and these paradoxical results interfered with dietary modification choices for PD patients. Therefore, based on comprehensive GWAS data of PUFAs and PD, our study utilized Mendelian randomization analyses to evaluate the causal relationship between omega-3 and omega-6 fatty acids and PD risk.

Our research found that the effect of AA on increased PD risk was statistically significant in Mendelian randomization. After we implemented MVMR and pleiotropic SNPs deletion to rule out the bias from other fatty acids, the result was still consistent. However, the weak odds ratio indicated limited causal effect and the need to further examine the underlying mechanisms involved. AA is an important precursor of multiple pro-inflammatory factors (prostaglandins-2, thromboxanes-2, and leukotrienes-3) and it is involved in the main inflammatory metabolic pathways, which could trigger oxidative response and furthermore damage the vulnerable substantia nigra cells (Bazinet and Layé, 2014; Janssen and Kiliaan, 2014; Ransohoff, 2016). However, the peripheral levels of AA analyzed in our study may not effectively reflect the cerebral inflammatory levels due to blood–brain barrier, and this may explain the limited effect on PD risk. Further studies in central levels of AA and PD risk are needed.

EPA was another possible risk factor for PD after MVMR adjustment, the bias may be explained by that half of the SNPs from EPA were also significantly associated with DPA. This non-causal effect could strongly dilute the risk from EPA. Indeed, EPA level was found to be significantly higher in PD patients compared with healthy controls in cerebral spinal fluid, while other PUFAs were below quantification limits (Lee et al., 2008). However, considering the small instrument sets of EPA, we recommend future verification of the result based on larger GWAS database.

No causal relationship was found between LA, DHA, DPA, ALA, and PD risk. Although sufficient power was guaranteed in LA, other omega-3 fatty acids exhibited limited power due to small instrument sets, and this may account for the null association. We relaxed the limitation threshold of *p-*value from 5E-08 up to 5E-06 to create larger instrument sets and implement debiased IVW to avoid bias from introducing weak instrumental variables into the analysis. Although the results were in accordance with the primary IVW ones, future larger GWAS database was still expected to acquire more reliable consequence. As for lifestyle intervention, Mediterranean diet rich in omega-3 fatty acids used to be regarded as a neuroprotective dietary pattern for aging population and for PD patients (Alcalay et al., 2012; Power et al., 2019; Metcalfe-Roach et al., 2021). Our results showed that it may not reduce the incidence of PD, and increased level of EPA may even be detrimental, though other nutrients in Mediterranean diet might be beneficial. Moreover, omega-3 fatty acids DHA was found to be moderately heterogeneous while its pleiotropic test showed no bias. We could infer that the heterogeneity most likely derived from database integration rather than pleiotropy (Hemani et al., 2018b).

Triangulation of our results with other researches was also significant in the interpretation of Mendelian randomization. One study applied polygenic risk score-based analysis to examine the potential genetic sharing between blood lipid levels and PD. Result indicated that AA had a positive concordance with PD, which was consistent with our results (Xicoy et al., 2021). Contrary to our conclusion, recent similar Mendelian randomization study stated that DHA was a protective factor for PD. Exploring probable reasons for the difference, we considered limited sample size and analyzing tools as the potential bias of the other study (Xue et al., 2021).

One limitation of our study was the limited strength of the odds ratio (OR = 1.03) between AA and PD risk, suggesting only a potential association and not a definite causal relationship. This weak causal relevance between peripheral levels of AA and PD risk indicated that further research was needed to fully understand the link between these factors. It is important to approach our findings with caution in clinical settings and to consider future studies that focus on increasing the sample size and validating our results. Additionally, it would be beneficial to investigate the impact of PUFAs on PD through the examination of cerebrospinal fluid levels. Another defect is about the generalizability of the study results. Differences between Mendelian randomization and randomized clinical trials existed. Exposures in Mendelian randomization had lifetime effect, which was different from the limited exposure duration in randomized controlled trials. The ability of Mendelian randomization to predict supplementary effects and instruct supplementary doses in randomized controlled trials was weak. In addition, our study focused on those who develop PD in later life, thus the estimates of PUFAs cannot be generalized to early-onset PD. Moreover, the European races of exposure and outcome data limited the generalizability of our result, so it was not appropriate to apply dietary modification suggested in the study to PD patients of other races. Additionally, it would be beneficial to conduct similar studies with diverse populations in order to confirm or refute the findings of this study. Furthermore, our research concentrated on six main PUFAs and discussed their causal relevance with PD, other types of PUFAs, like adrenic acid and dihomo-gamma-linolenic acid, may need further investigation with other more appropriate database.

Our study utilized Mendelian randomization to assess the causal relevance between PUFAs and PD risk. Results indicated AA as a risk factor and EPA as a possible risk factor for PD, while other PUFAs demonstrated no association. The causal relationship between PUFAs and PD risk was weak and had limited practical applications in clinical settings. Further research is needed.

## Data availability statement

The original contributions presented in the study are included in the article/Supplementary material, further inquiries can be directed to the corresponding author.

## Ethics statement

Written informed consent was obtained from the individual(s) for the publication of any potentially identifiable images or data included in this article.

## Author contributions

XZ contributed to the conceptualization and draft of the manuscript. SH contributed to the data analysis and draft of the manuscript. WK contributed to the revision of the manuscript. PC contributed to the data analysis. JL contributed to the supervision, conceptualization, review, and revision of the manuscript. All authors contributed to the article and approved the submitted version.

## Funding

This research was funded by National Natural Science Foundation of China, grant numbers 82230040 and 82071415; Shanghai Jiao Tong University Trans-med Awards Research, grant number20220103 (JL).

## Conflict of interest

The authors declare that the research was conducted in the absence of any commercial or financial relationships that could be construed as a potential conflict of interest.

## Publisher’s note

All claims expressed in this article are solely those of the authors and do not necessarily represent those of their affiliated organizations, or those of the publisher, the editors and the reviewers. Any product that may be evaluated in this article, or claim that may be made by its manufacturer, is not guaranteed or endorsed by the publisher.

## Footnotes

^1^ http://snipa.helmholtz-muenchen.de/snipa/index.php

^2^ http://www.phenoscanner.medschl.cam.ac.uk/
