# Supplementary material for: Associations between polyunsaturated fatty acid concentrations and Parkinson’s disease: A two-sample Mendelian randomization study
Source: Front Aging Neurosci. 2023 Feb 22;15:1123239. doi: 10.3389/fnagi.2023.1123239 (PMC9992541; doi:10.3389/fnagi.2023.1123239)
Supplement: Supplementary file 1 [file Table_1.DOCX]

Supplementary Material

Associations Between Polyunsaturated Fatty Acid Concentrations and Parkinson’s disease: a Two-sample Mendelian Randomization Study Supplementary Materials

Xue Zhu^1†^, Sijia Huang^1†^, Wenyan Kang^1^, Peizhan Chen^2^, Jun Liu^1, 3*^

*** Correspondence:** Jun Liu: [jly0520@hotmail.com](mailto:jly0520@hotmail.com)

# Supplementary Tables

| Phenotype | Sample size | Ancestry | Reference |
| --- | --- | --- | --- |
| Plasma levels of AA | 8631 participants | European | Guan et al.(Guan et al., 2014) |
| Plasma levels of LA | 13527 participants | European | Kettunen et al.(Kettunen et al., 2016) |
| Plasma levels of DHA, EPA, DPA and ALA | 8866 participants | European | Lemaitre et al.(Lemaitre et al., 2011) |
| Parkinson’s disease | 33674 cases and 449056 controls | European | Nalls et al.(Nalls et al., 2019) |

**Table S1. Data Sources.** AA, arachidonic acid; LA, linoleic acid; HDL, high density lipoprotein; TC, total cholesterol; TG, triglyceride; DHA, docosahexaenoic acid; EPA, eicosapentaenoic acid; DPA, docosapentaenoic acid; ALA, alpha-linolenic acid.

| Cohort | Number of participants | Mean age in  years (sd) | Female% | Measurement^*^ | Genotyping method | Quality control |
| --- | --- | --- | --- | --- | --- | --- |
| Cohorts used by Guan et al. | | | | | | |
| InCHIANTI | 1975 | 68.4（15.5） | 54.9 | gas chromatography  (plasma) | Illumina 550 | Call rate>97% H-W P>1*10^-4^ MAF>0.01 |
| ARIC | 3269 | 53.8（5.6） | 51.3 | thin layer plus gas chromatography  (plasma) | Affymetrix 6.0 | Call rate>95% H-W P>1*10^-6^ MAF>0.01 |
| CARDIA | 1507 | 45.8（3.4） | 53.1 |  | Affymetrix 6.0 | Call rate>95% H-W P>1*10^-4^ MAF>0.01 |
| CHS | 2404 | 72.0（5.1） | 61.6 |  | Illumina 370 | Call rate>97% H-W P>1*10^-5^ MAF>0.01 |
| MESA | 707 | 61.6（10.4） | 53.2 |  | Affymetrix 6.0 | Call rate>95% MAF>0.01 |
| Cohorts used by Kettunen et al. | | | | | | |
| EGCUT | 3287 | 46.3（19.5） | 58.0 | high-throughput nuclear magnetic resonance spectroscopy metabolomics  (plasma/serum) | 1000 Genomes imputation | NA |
| ERF | 2118 | 48.2（14.7） | 58.0 |  |  |  |
| FTC | 664 | 23.9（2.1） | 50.0 |  |  |  |
| FR97 | 3661 | 45.3（12.8） | 55.0 |  |  |  |
| COROGENE | 828 | 53.2（13.2） | 54.0 |  |  |  |
| GenMets | 572 | 55.8（7.3） | 57.0 |  |  |  |
| HBCS | 708 | 61.3（2.9） | 60.0 |  |  |  |
| KORA | 1745 | 60.9（8.8） | 52.0 |  |  |  |
| LLS | 2227 | 59.2（6.8） | 54.0 |  |  |  |
| NTR | 1192 | 38.8（12.8） | 64.0 |  |  |  |
| NFBC 1966 | 4709 | 31.2（0.4） | 51.0 |  |  |  |
| PredictCVD | 374 | 47.5（14.6） | 37.0 |  |  |  |
| PROTE | 597 | 38.3（16.0） | 51.0 |  |  |  |
| YFS | 2390 | 37.7（5.0） | 54.0 |  |  |  |
| Cohorts used by Lemaitre et al. | | | | | | |
| InCHIANTI | 1075 | 68.4（15.5） | 54.9 | gas chromatography  (plasma) | Illumina 550 | Call rate>97% H-W P>1*10^-4^ MAF>0.01 |
| ARIC | 3268 | 53.8（5.6） | 51.0 | thin layer plus gas chromatography  (plasma) | Affymetrix 6.0 | Call rate>95% H-W P>1*10^-6^ MAF>0.01 |
| CHS | 2326 | 72.0（5.1） | 61.3 |  | Illumina 370 | Call rate>97% H-W P>1*10^-5^ MAF>0.01 |
| CARDIA | 1507 | 45.8（3.4） | 53.3 |  | Affymetrix 6.0 | Call rate>95% H-W P>1*10^-4^ MAF>0.01 |
| MESA | 690 | 61.6（10.4） | 53.3 |  | Affymetrix 6.0 | Call rate>95% MAF>0.01 |

**Table S2. Study details for Exposure Data.** ARIC, Atherosclerosis Risk in Communities; CARDIA, Coronary Artery Risk Development in Young Adults; CHS, Cardiovascular Health Study; InCHIANTI, Invecchiare in Chianti; MESA, Ancillary study to the Multi-Ethnic Study of Atherosclerosis; COROGENE, Genetic Predisposition of Coronary Heart Disease in Patients Verified with Coronary Angiogram; EGCUT, Estonian Genome Center of University of Tartu Cohort; ERF, Erasmus Rucphen Family Study; FR97, a subsample of FINRISK 1997; FTC, Finnish Twin Cohort; GenMets, Genetics of METabolic Syndrome; HBCS, Helsinki Birth Cohort Study; KORA, Cooperative Health Research in the Region of Augsburg; LLS, Leiden Longevity Study; N, number of individuals with both genotype and metabolite traits analyzed; NFBC 1966, Northern Finland Birth Cohort 1966; NTR, Netherlands Twin Register; PredictCVD, FINRISK subsample of incident cardiovascular cases and controls; PROTE, EGCUT sub-cohort; YFS, The Cardiovascular Risk in Young Finns Study; H-W P, Hardy-Weinberg P; NA, not available. *Levels of PUFAs were quantified as percentage of total fatty acids.

|  |  |  |  |  |  |  |  | PUFA | | | PD | | | | |
| --- | --- | --- | --- | --- | --- | --- | --- | --- | --- | --- | --- | --- | --- | --- | --- |
|  | SNP | ea | nea | eaf | CHR | BP | gene | b | se | *P* | b | se | *P* | np | nc |
| AA | rs890455 | T | G | 0.0246 | 11 | 125259904 | PKNOX2 | 1.8507 | 0.3167 | 5.12E-09 | -0.0287 | 0.1208 | 0.8119 | 8166 | 6017 |
|  | rs7916429 | G | C | 0.0428 | 10 | 9705976 | HSP90AB7P | 0.5076 | 0.0893 | 1.32E-08 | -0.0222 | 0.0538 | 0.6795 | 26421 | 442271 |
|  | rs7456249 | C | T | 0.0185 | 7 | 149620802 | RP11-728K20.1 | 2.6799 | 0.4138 | 9.40E-11 | 0.1153 | 0.068 | 0.08989 | 27460 | 443025 |
|  | rs7231821 | T | A | 0.015 | 18 | 68738240 | RP11-529J17.1 | 0.8241 | 0.1295 | 1.99E-10 | 0.0187 | 0.0874 | 0.8306 | 26421 | 442271 |
|  | rs4359352 | A | C | 0.0257 | 14 | 20377949 | CTD-2335L22.6 | 2.4548 | 0.3579 | 6.97E-12 | 0.0131 | 0.0915 | 0.8862 | 27460 | 443025 |
|  | rs340480 | G | A | 0.0188 | 4 | 104380632 | RP11-119H12.3 | 2.9146 | 0.3928 | 1.17E-13 | 0.1932 | 0.0865 | 0.02557 | 7803 | 5852 |
|  | rs2967872 | C | G | 0.0317 | 16 | 84647733 | COTL1 | 1.6717 | 0.2987 | 2.20E-08 | 0.1487 | 0.0742 | 0.04514 | 7803 | 5852 |
|  | rs17663676 | C | T | 0.0341 | 11 | 62201002 | AHNAK | 0.4688 | 0.0857 | 4.52E-08 | 0.0532 | 0.0511 | 0.2976 | 33674 | 449056 |
|  | rs16951711 | C | T | 0.9867 | 13 | 96870398 | HS6ST3 | 1.8041 | 0.1274 | 1.56E-45 | -0.0107 | 0.0786 | 0.892 | 27823 | 443190 |
|  | rs16829840 | T | C | 0.014 | 3 | 119147958 | TMEM39A | 0.46 | 0.08 | 2.53E-08 | -0.0099 | 0.0565 | 0.8612 | 33674 | 449056 |
|  | rs12894905 | C | A | 0.9821 | 14 | 41497960 | RP11-129M6.1 | 0.9243 | 0.1105 | 5.91E-17 | -0.0938 | 0.0787 | 0.2337 | 26421 | 442271 |
|  | rs12796887 | G | A | 0.0208 | 11 | 10629295 | MRVI1 | 1.8843 | 0.331 | 1.26E-08 | 0.1299 | 0.0996 | 0.1923 | 25252 | 441303 |
|  | rs12747494 | C | T | 0.0128 | 1 | 91944137 | CDC7 | 2.6379 | 0.4088 | 1.10E-10 | 0.0626 | 0.0521 | 0.2297 | 27823 | 443190 |
|  | rs12416578 | C | T | 0.0105 | 10 | 68212659 | CTNNA3 | 0.9577 | 0.1242 | 1.28E-14 | 0.0142 | 0.1068 | 0.8942 | 26421 | 442271 |
|  | rs11927316 | A | T | 0.0174 | 3 | 133893303 | RYK | 0.7118 | 0.1301 | 4.52E-08 | 0.0074 | 0.084 | 0.9296 | 26421 | 442271 |
|  | rs11726352 | A | G | 0.0144 | 4 | 25874805 | SMIM20 | 2.6898 | 0.4275 | 3.14E-10 | 0.1313 | 0.0814 | 0.1066 | 26421 | 442271 |
|  | rs11578575 | T | C | 0.0222 | 1 | 102489339 | OLFM3 | 2.5538 | 0.3582 | 1.00E-12 | 0.0124 | 0.0963 | 0.8977 | 26421 | 442271 |
|  | rs10788947 | A | G | 0.0481 | 1 | 53513168 | SCP2 | 0.5477 | 0.0805 | 1.01E-11 | 0.0267 | 0.0384 | 0.4876 | 33674 | 449056 |
|  | rs10488885 | T | C | 0.0163 | 4 | 110584034 | MCUB | 2.2838 | 0.3908 | 5.10E-09 | 0.0838 | 0.0789 | 0.2885 | 26421 | 442271 |
|  | rs9356335* | G | A | 0.0216 | 6 | 165413927 | XX-C2158C12.1 | 1.1577 | 0.0984 | 6.06E-32 | -0.0413 | 0.0722 | 0.5669 | 26421 | 442271 |
|  | rs7970058* | G | A | 0.9898 | 12 | 12988199 | TMEM132D | 0.8683 | 0.1117 | 7.68E-15 | 0.0659 | 0.1007 | 0.5127 | 26421 | 442271 |
|  | rs7546429* | A | G | 0.9758 | 1 | 118028688 | MAN1A2 | 2.7454 | 0.1467 | 3.98E-78 | 0.0913 | 0.0729 | 0.2105 | 33147 | 448584 |
|  | rs7137292* | A | G | 0.984 | 12 | 33866915 | RP13-359K18.1 | 0.6337 | 0.1091 | 6.38E-09 | 0.0572 | 0.0581 | 0.3252 | 33674 | 449056 |
|  | rs4072288* | G | A | 0.981 | 14 | 104889069 | RNU6-684P | 2.1978 | 0.4022 | 4.64E-08 | 0.0087 | 0.0798 | 0.9128 | 26421 | 442271 |
|  | rs2653765* | C | A | 0.9866 | 12 | 38998940 | RP11-804F13.1 | 1.2721 | 0.1353 | 5.47E-21 | -0.0042 | 0.094 | 0.9647 | 13887 | 11669 |
|  | rs2294281* | G | C | 0.9869 | 6 | 116223376 | FRK | 1.039 | 0.1207 | 7.58E-18 | 0.0587 | 0.0906 | 0.5173 | 27460 | 443025 |
|  | rs2072114* | A | G | 0.8817 | 11 | 61605215 | FADS2 | 1.5622 | 0.0382 | 1.00E-200 | 0.0332 | 0.0254 | 0.1917 | 33674 | 449056 |
|  | rs17811780* | G | T | 0.0189 | 18 | 30030309 | GAREM1 | 0.6849 | 0.12 | 1.16E-08 | 0.0607 | 0.0646 | 0.3472 | 33674 | 449056 |
|  | rs1741* | G | C | 0.6921 | 16 | 15130351 | PDXDC1 | 0.2008 | 0.0314 | 1.64E-10 | -0.0155 | 0.0184 | 0.3982 | 33674 | 449056 |
|  | rs13314643* | T | C | 0.0124 | 3 | 41139829 | RP11-944L7.4 | 1.4368 | 0.1531 | 6.32E-21 | -0.0099 | 0.0838 | 0.906 | 26421 | 442271 |
|  | rs11147144* | A | C | 0.0117 | 12 | 133462329 | CHFR | 0.8943 | 0.1269 | 1.85E-12 | 0.0238 | 0.0698 | 0.7334 | 33674 | 449056 |
| LA | rs9341222 | C | T | 0.019 | 2 | 217529410 | IGFBP2 | 5.1228 | 0.531 | 5.04E-22 | -0.0115 | 0.0882 | 0.8963 | 26421 | 442271 |
|  | rs871900 | A | G | 0.0117 | 19 | 32621441 | AC011518.2 | 1.2985 | 0.1887 | 5.95E-12 | 0.0955 | 0.0843 | 0.2569 | 26421 | 442271 |
|  | rs8176902 | C | T | 0.0227 | 12 | 79990210 | PAWR | 3.7383 | 0.478 | 5.24E-15 | 0.0935 | 0.1332 | 0.483 | 22756 | 439477 |
|  | rs8031736 | C | A | 0.0114 | 15 | 64551588 | CSNK1G1 | 4.711 | 0.6959 | 1.29E-11 | 0.0494 | 0.0879 | 0.5746 | 26421 | 442271 |
|  | rs7847397 | G | A | 0.0298 | 9 | 132133022 | RN7SL159P | 1.9996 | 0.3371 | 3.01E-09 | 0.0388 | 0.0655 | 0.5532 | 26421 | 442271 |
|  | rs7789451 | G | A | 0.9879 | 7 | 24002316 | AC009508.1 | 1.1888 | 0.1781 | 2.50E-11 | -0.0101 | 0.0738 | 0.8906 | 26421 | 442271 |
|  | rs7786525 | A | C | 0.055 | 7 | 4763157 | FOXK1 | 1.4899 | 0.2222 | 2.02E-11 | -0.0203 | 0.0316 | 0.5192 | 26421 | 442271 |
|  | rs7681918 | T | C | 0.0194 | 4 | 63475589 | DPP3P1 | 3.0489 | 0.5104 | 2.33E-09 | 0.0941 | 0.0889 | 0.2898 | 26421 | 442271 |
|  | rs7681753 | C | G | 0.9892 | 4 | 54304925 | RP11-231C18.3 | 1.19 | 0.2146 | 2.80E-08 | -0.2526 | 0.1264 | 0.04567 | 6634 | 4884 |
|  | rs7478695 | T | C | 0.0153 | 11 | 133584361 | RP11-448P19.1 | 3.5508 | 0.5906 | 1.83E-09 | -0.0502 | 0.0674 | 0.4562 | 26421 | 442271 |
|  | rs7214940 | T | C | 0.0178 | 17 | 69557111 | AC118653.2 | 3.2319 | 0.5542 | 5.50E-09 | -0.1393 | 0.155 | 0.3688 | 25252 | 441303 |
|  | rs6668309 | C | G | 0.019 | 1 | 2214587 | SKI | 3.1048 | 0.4972 | 4.26E-10 | 0.0245 | 0.0439 | 0.5765 | 26421 | 442271 |
|  | rs656116 | C | G | 0.0268 | 18 | 8721372 | MTCL1 | 2.291 | 0.3866 | 3.09E-09 | -0.005 | 0.0822 | 0.9515 | 26421 | 442271 |
|  | rs4724375 | T | C | 0.0347 | 7 | 45243393 | RAMP3 | 2.03 | 0.3646 | 2.59E-08 | -0.067 | 0.0863 | 0.4376 | 7803 | 5852 |
|  | rs4488492 | C | G | 0.0543 | 17 | 9467552 | STX8 | 2.0372 | 0.3137 | 8.42E-11 | -0.0618 | 0.0634 | 0.3302 | 7803 | 5852 |
|  | rs4245549 | A | C | 0.0179 | 7 | 50603534 | FIGNL1 | 1.2731 | 0.1813 | 2.18E-12 | 0.1061 | 0.0596 | 0.07501 | 33288 | 448563 |
|  | rs2518519 | A | G | 0.0255 | 1 | 158845728 | PYHIN5P | 2.5693 | 0.4515 | 1.27E-08 | 0.0446 | 0.0229 | 0.05164 | 33674 | 449056 |
|  | rs2516069 | T | C | 0.0144 | 22 | 27393302 | ISX | 3.3835 | 0.5506 | 7.98E-10 | -0.014 | 0.0855 | 0.8701 | 26421 | 442271 |
|  | rs2306211 | A | G | 0.0225 | 3 | 121151784 | POLQ | 1.0884 | 0.1703 | 1.65E-10 | 0.0798 | 0.0642 | 0.2136 | 33674 | 449056 |
|  | rs2232720 | T | G | 0.0399 | 1 | 28563008 | ATPIF1 | 2.1543 | 0.3693 | 5.42E-09 | -0.2399 | 0.0793 | 0.002473 | 26291 | 442057 |
|  | rs17821896 | G | C | 0.01 | 16 | 80516614 | RP11-525K10.3 | 4.5626 | 0.6928 | 4.52E-11 | 0.021 | 0.0408 | 0.6067 | 26421 | 442271 |
|  | rs17758255 | C | T | 0.0452 | 17 | 53787963 | TMEM100 | 2.1662 | 0.3481 | 4.87E-10 | 0.0314 | 0.0691 | 0.6497 | 26421 | 442271 |
|  | rs17092999 | T | C | 0.9743 | 20 | 34298096 | RBM39 | 1.0616 | 0.1417 | 6.84E-14 | -0.0305 | 0.0474 | 0.5201 | 33674 | 449056 |
|  | rs13082565 | T | C | 0.0349 | 3 | 77095077 | ROBO2 | 2.4974 | 0.3929 | 2.07E-10 | 0.1269 | 0.0969 | 0.1902 | 26421 | 442271 |
|  | rs12923143 | C | T | 0.016 | 16 | 1587067 | IFT140 | 4.3017 | 0.5833 | 1.65E-13 | 0.0588 | 0.0796 | 0.4602 | 33311 | 448891 |
|  | rs1245908 | G | C | 0.0152 | 10 | 111529233 | RPL21P91 | 3.7134 | 0.6397 | 6.44E-09 | 0.0207 | 0.0796 | 0.7947 | 26421 | 442271 |
|  | rs12425981 | T | C | 0.0183 | 12 | 1962113 | CACNA2D4 | 3.145 | 0.5534 | 1.32E-08 | 0.0533 | 0.0941 | 0.5708 | 26421 | 442271 |
|  | rs12403588 | T | G | 0.0106 | 1 | 106417259 | RP11-90H3.2 | 3.763 | 0.6661 | 1.61E-08 | 0.0401 | 0.0368 | 0.276 | 26421 | 442271 |
|  | rs12190460 | A | G | 0.0277 | 6 | 3881764 | TMEM14C | 2.7379 | 0.4446 | 7.38E-10 | -0.1176 | 0.0595 | 0.04809 | 7803 | 5852 |
|  | rs11755506 | C | A | 0.013 | 6 | 106084237 | RP3-359N14.2 | 1.7756 | 0.1899 | 8.74E-21 | -0.0336 | 0.0739 | 0.6489 | 26421 | 442271 |
|  | rs11564738 | T | C | 0.0139 | 11 | 2020504 | H19 | 3.4806 | 0.6244 | 2.49E-08 | -0.0994 | 0.1179 | 0.3989 | 8315 | 6134 |
|  | rs10757497 | T | C | 0.0118 | 9 | 24903603 | RMRPP5 | 3.9375 | 0.6527 | 1.62E-09 | -0.0027 | 0.0242 | 0.9098 | 26421 | 442271 |
|  | rs10752382 | A | C | 0.0267 | 10 | 15523805 | ITGA8 | 2.5711 | 0.4706 | 4.67E-08 | -0.0152 | 0.0223 | 0.4962 | 26421 | 442271 |
|  | rs10474568 | T | G | 0.0192 | 5 | 78106262 | ARSB | 3.0814 | 0.5326 | 7.22E-09 | -0.0189 | 0.1412 | 0.8937 | 7161 | 5356 |
|  | rs10454501 | C | T | 0.0121 | 12 | 115593866 | RP11-25E2.1 | 3.7487 | 0.6689 | 2.09E-08 | -0.025 | 0.0343 | 0.4672 | 27296 | 442718 |
|  | rs10072644 | T | C | 0.0117 | 5 | 5646774 | LINC02142 | 1.0706 | 0.1901 | 1.80E-08 | -0.1269 | 0.1066 | 0.2339 | 25252 | 441303 |
|  | rs7080386 | C | A | 0.5674 | 10 | 65048306 | JMJD1C | 0.254 | 0.0438 | 6.80E-09 | 0.0084 | 0.0171 | 0.6232 | 33674 | 449056 |
|  | rs10189621* | T | G | 0.9874 | 2 | 197311221 | HECW2 | 2.3755 | 0.1692 | 9.03E-45 | 0.1048 | 0.1009 | 0.2987 | 25252 | 441303 |
|  | rs11147144* | C | A | 0.9886 | 12 | 133462329 | CHFR | 1.1193 | 0.1696 | 4.12E-11 | 0.0238 | 0.0698 | 0.7334 | 33674 | 449056 |
|  | rs1136001* | T | G | 0.3094 | 16 | 15131974 | NTAN1 | 0.3521 | 0.0439 | 9.86E-16 | -0.0175 | 0.0183 | 0.3411 | 33674 | 449056 |
|  | rs11967152* | C | A | 0.0143 | 6 | 135844014 | LINC00271 | 2.0017 | 0.2088 | 9.07E-22 | 0.0942 | 0.0796 | 0.2366 | 26421 | 442271 |
|  | rs12755498* | T | G | 0.0117 | 1 | 56942680 | PLPP3 | 3.7647 | 0.6585 | 1.09E-08 | -0.0164 | 0.02 | 0.4117 | 33674 | 449056 |
|  | rs13114065* | T | C | 0.0121 | 4 | 59917164 | RP11-340A13.3 | 1.0603 | 0.1924 | 3.58E-08 | 0.0894 | 0.0875 | 0.307 | 26421 | 442271 |
|  | rs13314643* | C | T | 0.9883 | 3 | 41139829 | RP11-944L7.4 | 1.9089 | 0.1792 | 1.65E-26 | -0.0099 | 0.0838 | 0.906 | 26421 | 442271 |
|  | rs16974991* | A | G | 0.0106 | 15 | 95814539 | LINC01197 | 2.0761 | 0.2708 | 1.77E-14 | 0.1228 | 0.1562 | 0.432 | 6634 | 4884 |
|  | rs174528* | C | T | 0.3626 | 11 | 61543499 | MYRF | 1.4342 | 0.0425 | 1.00E-200 | -0.006 | 0.0179 | 0.7365 | 33674 | 449056 |
|  | rs17811780* | T | G | 0.9809 | 18 | 30030309 | GAREM1 | 1.3034 | 0.169 | 1.25E-14 | 0.0607 | 0.0646 | 0.3472 | 33674 | 449056 |
|  | rs2294281* | C | G | 0.0103 | 6 | 116223376 | FRK | 2.2143 | 0.183 | 1.05E-33 | -0.0587 | 0.0906 | 0.5173 | 27460 | 443025 |
|  | rs4072288* | A | G | 0.019 | 14 | 104889069 | RNU6-684P | 4.1429 | 0.5446 | 2.81E-14 | 0.0087 | 0.0798 | 0.9128 | 26421 | 442271 |
|  | rs4912642* | G | C | 0.012 | 5 | 141931618 | AC005592.2 | 1.1 | 0.1901 | 7.23E-09 | -0.0113 | 0.0824 | 0.8913 | 26421 | 442271 |
|  | rs6083308* | G | T | 0.9792 | 20 | 23839581 | CFTRP3 | 1.3791 | 0.2042 | 1.44E-11 | 0.0377 | 0.0274 | 0.1689 | 26421 | 442271 |
|  | rs6122806* | A | C | 0.0148 | 20 | 48289052 | B4GALT5 | 1.1861 | 0.184 | 1.14E-10 | 0.0671 | 0.0684 | 0.326 | 33674 | 449056 |
|  | rs7137292* | G | A | 0.0212 | 12 | 33866915 | RP13-359K18.1 | 1.7045 | 0.1627 | 1.13E-25 | 0.0572 | 0.0581 | 0.3252 | 33674 | 449056 |
|  | rs7546429* | G | A | 0.0207 | 1 | 118028688 | MAN1A2 | 5.2311 | 0.2261 | 2.22E-118 | 0.0913 | 0.0729 | 0.2105 | 33147 | 448584 |
| DHA | rs143988316 | C | T | 0.930513 | 19 | 19667254 | PBX4 | 0.150045 | 0.024351 | 1.10E-09 | -0.0287 | 0.032 | 0.3712 | 33674 | 449056 |
|  | rs261334* | G | C | 0.230871 | 15 | 58726744 | LIPC | 0.110247 | 0.014749 | 1.44E-13 | 0.0222 | 0.0216 | 3.03E-01 | 33674 | 449056 |
|  | rs11604424* | C | T | 0.243328 | 11 | 116651115 | ZPR1 | 0.08311 | 0.014241 | 7.84E-09 | -0.0394 | 0.0206 | 5.63E-02 | 33674 | 449056 |
|  | rs2281591* | A | G | 0.86628 | 6 | 10990493 | ELOVL2 | 0.108394 | 0.018174 | 3.66E-09 | 0.0311 | 0.0198 | 1.16E-01 | 33674 | 449056 |
|  | rs174546* | C | T | 0.597151 | 11 | 61569830 | FADS1 | 0.127635 | 0.012483 | 4.81E-24 | 0.0029 | 0.0179 | 8.73E-01 | 33674 | 449056 |
|  | rs145717049 | C | T | 0.955942 | 19 | 19130096 | SUGP2 | 0.201292 | 0.03275 | 1.21E-09 | -0.2469 | 0.1439 | 0.08632 | 6634 | 4884 |
| EPA | rs174538* | G | A | 0.7196 | 11 | 61560081 | TMEM258 | 0.0834 | 0.0052 | 5.37E-58 | 0.013 | 0.0183 | 0.4801 | 33674 | 449056 |
|  | rs3798713* | C | G | 0.2322 | 6 | 11008622 | ELOVL2 | 0.035 | 0.005 | 1.93E-12 | -0.0051 | 0.0169 | 0.7654 | 33674 | 449056 |
|  | rs7875586 | T | C | 0.0428 | 9 | 95891998 | NINJ1 | 0.4228 | 0.0698 | 1.40E-09 | 0.0207 | 0.0517 | 0.6895 | 33674 | 449056 |
|  | rs1191103 | C | G | 0.1256 | 14 | 100355196 | EML1 | 0.2323 | 0.0411 | 1.62E-08 | 0.0021 | 0.0349 | 0.9512 | 26421 | 442271 |
| DPA | rs3734398* | T | C | 0.5677 | 6 | 10982973 | EML1 | 0.04 | 0.003 | 9.61E-44 | -5.00E-04 | 0.0169 | 0.9774 | 33674 | 449056 |
|  | rs174547* | T | C | 0.6717 | 11 | 61570783 | FADS1 | 0.075 | 0.003 | 3.79E-154 | -0.0033 | 0.0179 | 0.8525 | 33674 | 449056 |
| ALA | rs174547* | T | C | 0.6717 | 11 | 61570783 | FADS1 | 0.016 | 0.001 | 3.47E-64 | 0.0033 | 0.0179 | 0.8525 | 33674 | 449056 |

**Table S3. Polyunsaturated Fatty Acid Instruments Used for Mendelian Randomization Analyses.** PUFA, polyunsaturated fatty acid; PD, Parkinson’s disease; SNP, single nucleotide polymorphism; ea, effect allele; nea, non-effect allele; eaf, effect allele frequency; b, beta; se, standard error; CHR, chromosome; POS, position; AA, arachidonic acid; LA, linoleic acid; DHA, docosahexaenoic acid; EPA, eicosapentaenoic acid; DPA, docosapentaenoic acid; ALA, alpha-linolenic acid. * Pleiotropic SNPs searched through Phenoscanner.

|  | Cases | | | | Controls | | | |  |  |
| --- | --- | --- | --- | --- | --- | --- | --- | --- | --- | --- |
| Cohort | Number of participants | Female% | Mean age in years (sd) | Diagnostic criteria | Number of participants | Female% | Mean age in years (sd) | Diagnostic criteria | Array type | Quality control |
| Finnish Parkinson's | 386 | 45.85 | 55.27 (5.64) | Clinic visit, standard UK Brain Bank criteria with a modification to allow the inclusion of cases that had a family history of PD | 493 | 78.9 | 92.35 (3.86) | Population control | GWAS array | Call rate>95% H-W P>1*10^-4^ MAF>0.001 |
| Baylor College of Medicine / University of Maryland | 769 | 33.81 | 64.83 (10.12) |  | 195 | 69.74 | 65.48 (8.31) | Clinic visit and self-report |  |  |
| McGill Parkinson's | 582 | 34.54 | 65.71 (9.79) |  | 905 | 48.4 | 55.79 (10.69) |  |  |  |
| OPDS | 476 | 35.71 | 65.32 (9.28) |  | 462 | 42.21 | 61.85 (11.06) |  |  |  |
| Spanish Parkinson's (IPDGC) | 2110 | 43.13 | 63.92 (12.54) |  | 1333 | 54.39 | 64.03 (12.59) |  |  |  |
| Tubingen Parkinson's Disease cohort | 666 | 36.04 | 59.89 (11.25) |  | 542 | 57.93 | 67.48 (8.41) |  |  |  |
| Vance | 620 | 27.74 | 77.47 (8.40) |  | 299 | 50.84 | 81.98 (12.78) |  |  |  |
| UK PDMED | 1025 | 32.78 | NA |  | 655 | 72.67 | NA |  |  |  |
| PDBP | 512 | 38.67 | 64.46 (9.37) |  | 282 | 51.06 | 62.19 (10.73) |  | targeted, NeuroX array |  |
| PPMI | 363 | 33.06 | 64.24 (9.65) |  | 165 | 33.33 | 63.79 (10.59) |  |  |  |
| HBS | 527 | 34.35 | 66.31 (10.07) |  | 472 | 61.65 | 69.9 (9.02) |  |  |  |
| NeuroX | 5851 | NA | NA |  | 5866 | NA | NA |  |  |  |
| SGPD | 1169 | 35.24 | 59.88 (10.86) |  | 968 | 53.93 | 66.64 (9.65) |  | GWAS array | Call rate>95% H-W P>1*10^-6^ MAF>0.01 |
| UK BioBank | 18618 | 57.62 | 58.45 (7.20) | defined by family history | 436419 | 54.14 | 56.69 (8.05) | No ICD-10 codes for PD or family history of PD |  | NA |

**Table S4. Study Details for Outcome Data.** OPDS, Oslo Parkinson's Disease Study; Vance, Vance (dbGap phs000394); PDBP, Parkinson's Disease Biomarker's Program; PPMI, Parkinson's Progression Markers Initiative; HBS, Harvard Biomarker Study, NeuroX, NeuroX - dbGaP (phs000918.v1.p1); SGPD, System Genomics of Parkinson's Disease; NA, not available.

| PUFAs | SNP | Other Phenotypes | *P* |
| --- | --- | --- | --- |
| AA | rs9356335 | Dihomo-gamma-linolenic acid | 6.45E-09 |
|  |  | Gamma-linolenic acid | 4.45E-12 |
|  | rs7970058 | Linoleic acid | 1.56E-09 |
|  | rs7546429 | Dihomo-gamma-linolenic acid | 5.18E-26 |
|  |  | Gamma-linolenic acid | 4.77E-72 |
|  |  | Linoleic acid | 2.22E-118 |
|  | rs7137292 | Linoleic acid | 1.13E-25 |
|  | rs4072288 | Linoleic acid | 2.81E-14 |
|  | rs2653765 | Gamma-linolenic acid | 9.29E-17 |
|  | rs2294281 | Dihomo-gamma-linolenic acid | 8.40E-28 |
|  |  | Linoleic acid | 1.05E-33 |
|  | rs2072114 | Alpha-linolenic acid | 2.41E-28 |
|  |  | Adrenic acid | 6.04E-59 |
|  |  | Dihomo-gamma-linolenic acid | 5.95E-15 |
|  |  | Gamma-linolenic acid | 2.34E-31 |
|  |  | Linoleic acid | 6.13E-141 |
|  |  | Low density lipoprotein | 2.47E-17 |
|  |  | Total cholesterol | 2.35E-18 |
|  |  | Triglycerides | 5.25E-20 |
|  |  | High density lipoprotein | 1.23E-14 |
|  |  | Docosapentaenoic acid | 4.45E-64 |
|  |  | Eicosapentaenoic acid | 1.42E-28 |
|  | rs17811780 | Gamma-linolenic acid | 5.26E-13 |
|  |  | Linoleic acid | 1.25E-14 |
|  | rs1741 | Dihomo-gamma-linolenic acid | 5.17E-65 |
|  |  | Gamma-linolenic acid | 3.09E-11 |
|  |  | Linoleic acid | 1.00E-15 |
|  | rs13314643 | Linoleic acid | 1.65E-26 |
|  | rs11147144 | Linoleic acid | 4.12E-11 |
| LA | rs10189621 | Dihomo-gamma-linolenic acid | 4.39E-08 |
|  | rs1136001 | Arachidonic acid | 2.10E-10 |
|  |  | Dihomo-gamma-linolenic acid | 3.66E-65 |
|  |  | Gamma-linolenic acid | 2.88E-11 |
|  | rs11967152 | Dihomo-gamma-linolenic acid | 4.09E-10 |
|  | rs12755498 | Gamma-linolenic acid | 1.36E-09 |
|  | rs13114065 | Gamma-linolenic acid | 2.04E-08 |
|  | rs16974991 | Arachidonic acid | 1.57E-17 |
|  | rs17811780 | Arachidonic acid | 1.16E-08 |
|  |  | Gamma-linolenic acid | 5.26E-13 |
|  | rs2294281 | Arachidonic acid | 7.58E-18 |
|  |  | Dihomo-gamma-linolenic acid | 8.40E-28 |
|  | rs4072288 | Arachidonic acid | 4.64E-08 |
|  | rs4912642 | Gamma-linolenic acid | 2.30E-11 |
|  | rs6083308 | Dihomo-gamma-linolenic acid | 1.12E-61 |
|  |  | Gamma-linolenic acid | 3.64E-11 |
|  | rs6122806 | Dihomo-gamma-linolenic acid | 1.78E-14 |
|  | rs7137292 | Arachidonic acid | 6.38E-09 |
|  | rs7546429 | Arachidonic acid | 3.98E-78 |
|  |  | Dihomo-gamma-linolenic acid | 5.18E-26 |
|  |  | Gamma-linolenic acid | 4.77E-72 |
| DHA | rs261334 | High density lipoprotein | 8.58E-71 |
|  |  | Total cholesterol | 1.46E-15 |
|  |  | Triglycerides | 3.95E-13 |
|  | rs11604424 | High density lipoprotein | 1.22E-17 |
|  |  | Low density lipoprotein | 4.70E-11 |
|  |  | Total cholesterol | 1.45E-29 |
|  |  | Triglycerides | 1.65E-141 |
|  | rs2281591 | Docosapentaenoic acid | 1.58E-38 |
|  |  | Eicosapentaenoic acid | 1.41E-09 |
| EPA | rs3798713 | Docosahexaenoic acid | 1.40E-15 |
|  |  | Docosapentaenoic acid | 9.71E-43 |
| DPA | rs3734398 | Docosahexaenoic acid | 1.65E-15 |
|  |  | Eicosapentaenoic acid | 3.99E-12 |
| AA/LA/DHA/EPA  /DPA/ALA | rs174547* | Dihomo-gamma-linolenic acid | 2.63E-151 |
|  |  | Gamma-linolenic acid | 2.29E-72 |
|  |  | High density lipoprotein | 4.05E-27 |
|  |  | Low density lipoprotein | 7.99E-38 |
|  |  | Total cholesterol | 1.35E-35 |
|  |  | Triglycerides | 1.04E-40 |

**Table S5. Pleiotropic SNPs of PUFAs searched through Phenoscanner.** PUFA, polyunsaturated fatty acid; SNP, single nucleotide polymorphism; AA, arachidonic acid; LA, linoleic acid; DHA, docosahexaenoic acid; EPA, eicosapentaenoic acid; DPA, docosapentaenoic acid; ALA, alpha-linolenic acid. * rs174528, rs174538 and rs174546 were in linkage disequilibrium with rs174547, so their phenotypes were summarized together with rs174547.

# Supplementary Figures


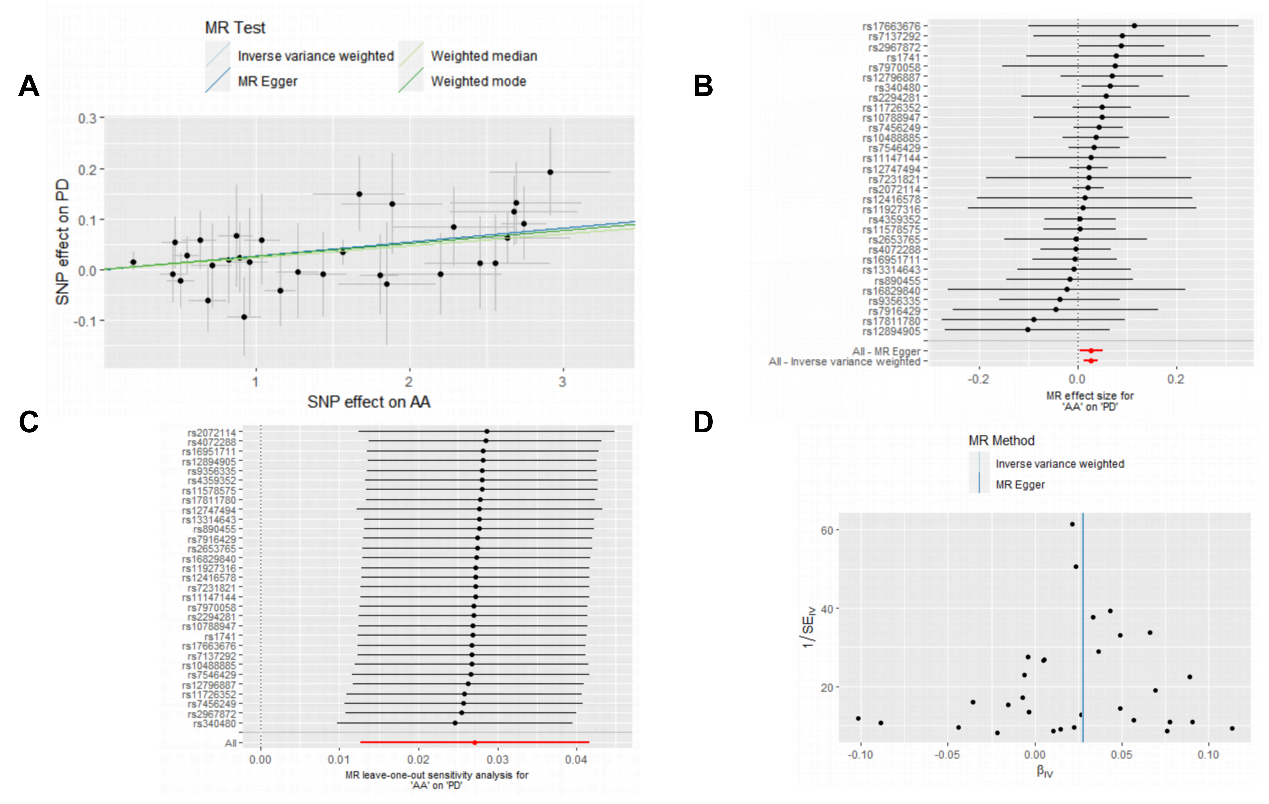


**Figure S1. Mendelian Randomization (MR) Sensitivity Plots of the Causal Effect of Arachidonic Acid (AA) Levels on Parkinson’s Disease.** A) Scatter plot showing results of the 4 MR mendelian randomization methods, B) Forest plot showing individual single nucleotide polymorphism (SNP) ratio estimates (SNP-outcome effect estimate / SNP-exposure effect estimate), C) Leave-one-out plot showing inverse variance weighted (IVW) estimates after omitting each SNP, and D) Funnel plot showing instrument precision: ratio estimate in log (odds ratio) (βIV; x-axis) by instrument strength (1/ βIV standard error [SE]). Asymmetry in this plot may be a result of horizontal pleiotropy.


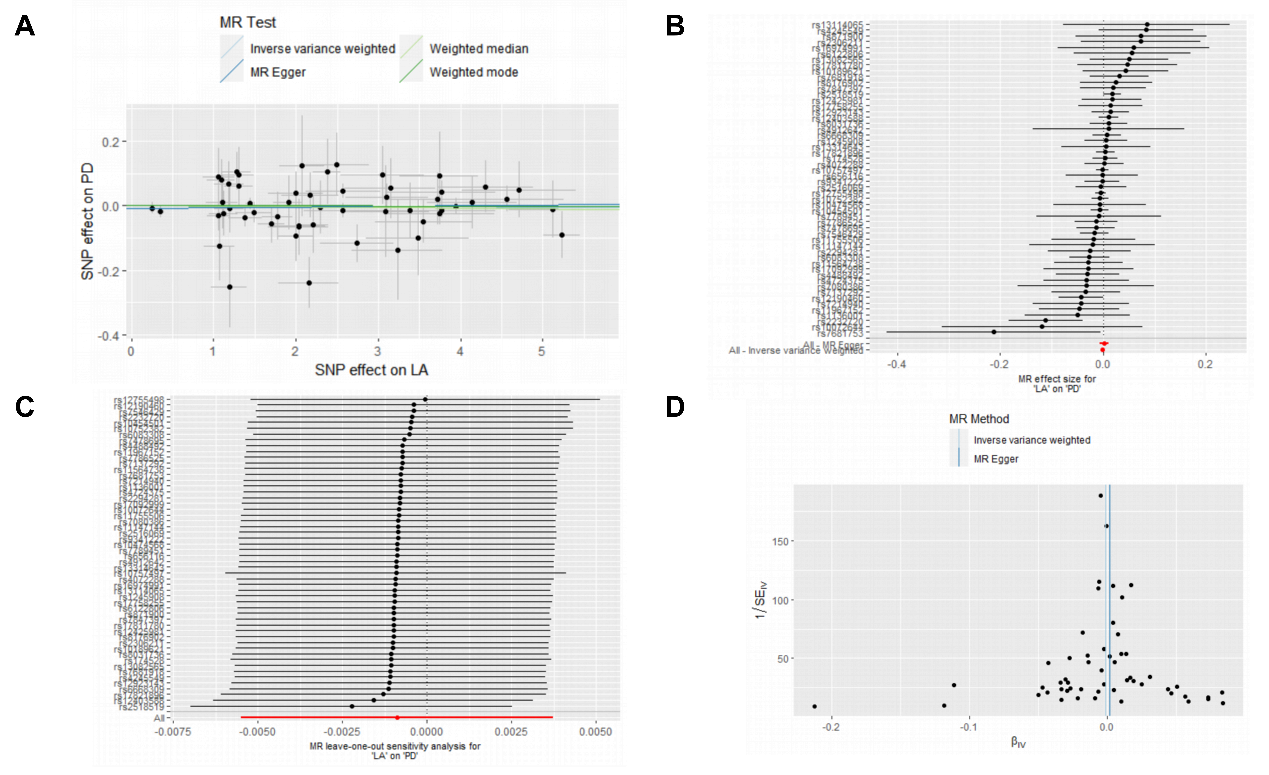


**Figure S2. Mendelian Randomization (MR) Sensitivity Plots of the Causal Effect of Linoleic Acid (LA) Levels on Parkinson’s Disease.** A) Scatter plot showing results of the 4 MR mendelian randomization methods, B) Forest plot showing individual single nucleotide polymorphism (SNP) ratio estimates (SNP-outcome effect estimate / SNP-exposure effect estimate), C) Leave-one-out plot showing inverse variance weighted (IVW) estimates after omitting each SNP, and D) Funnel plot showing instrument precision: ratio estimate in log (odds ratio) (βIV; x-axis) by instrument strength (1/ βIV standard error [SE]). Asymmetry in this plot may be a result of horizontal pleiotropy.


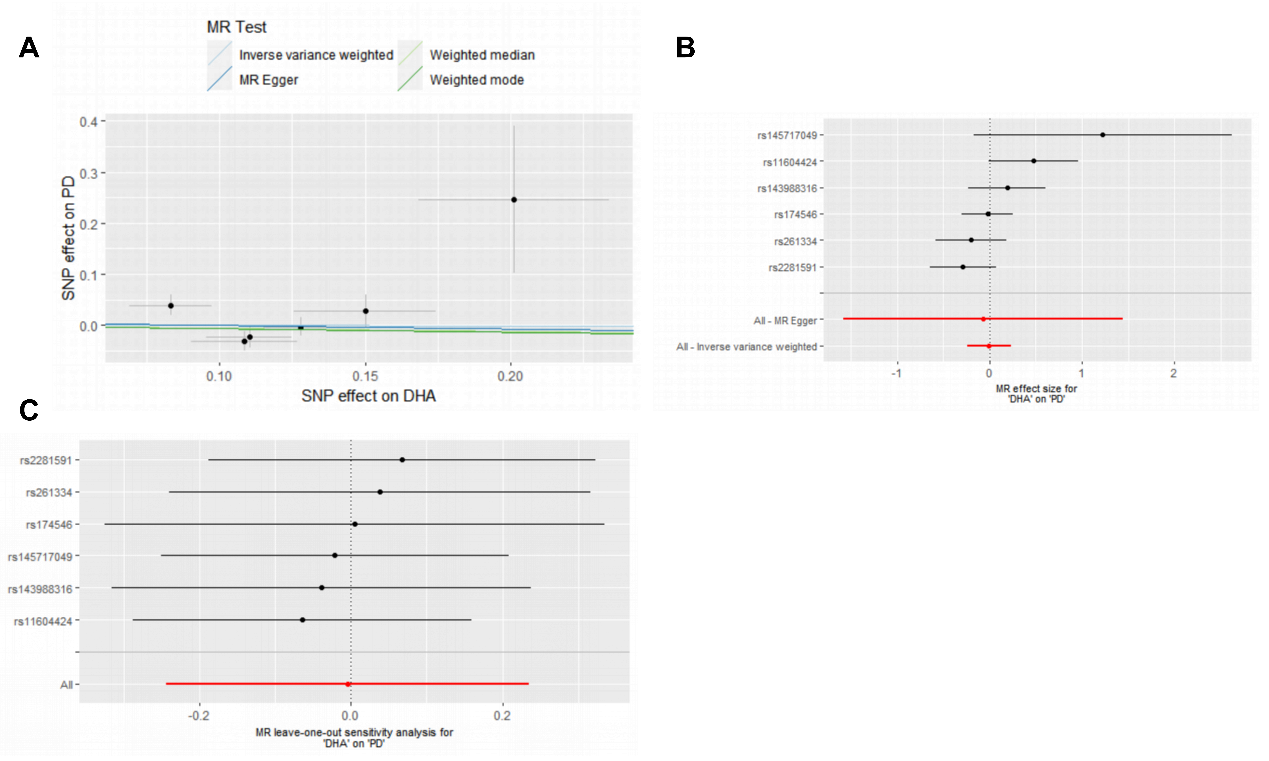


**Figure S3. Mendelian Randomization (MR) Sensitivity Plots of the Causal Effect of Docosahexaenoic Acid (DHA) Levels on Parkinson’s Disease.** A) Scatter plot showing results of the 4 MR mendelian randomization methods, B) Forest plot showing individual single nucleotide polymorphism (SNP) ratio estimates (SNP-outcome effect estimate / SNP-exposure effect estimate), and C) Leave-one-out plot showing inverse variance weighted (IVW) estimates after omitting each SNP.


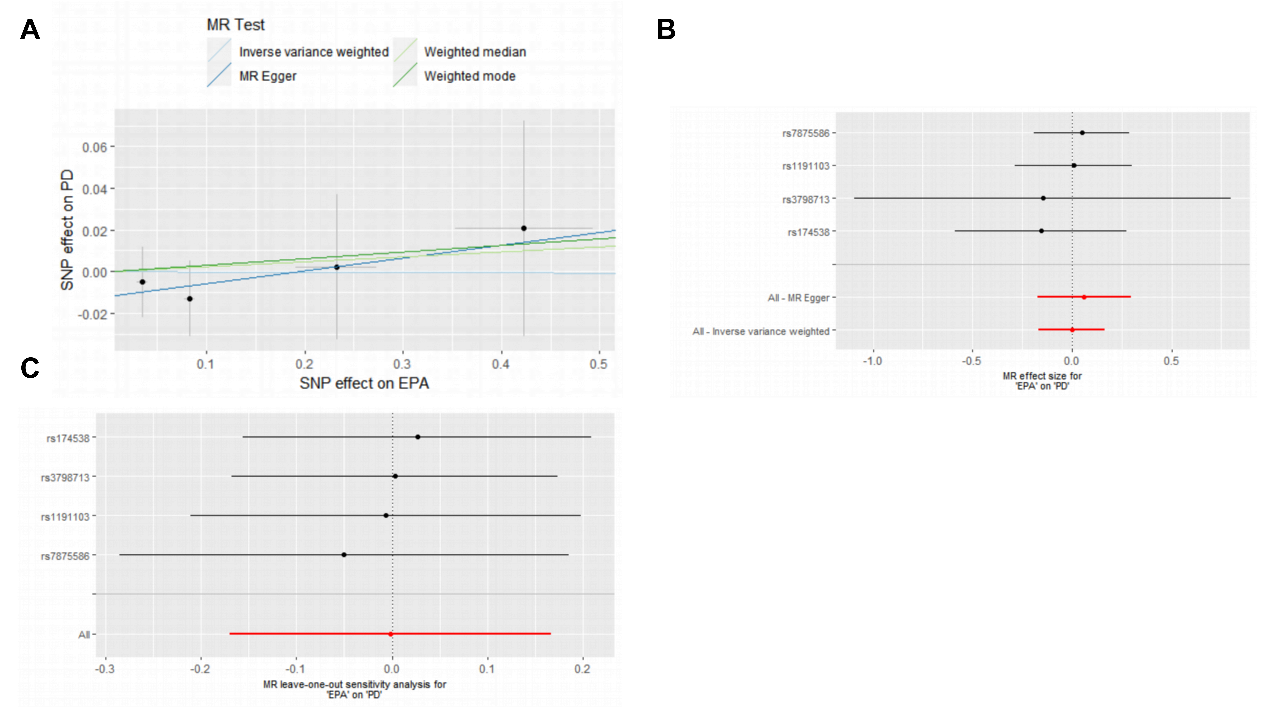


**Figure S4. Mendelian Randomization (MR) Sensitivity Plots of the Causal Effect of Eicosapentaenoic Acid (EPA) Levels on Parkinson’s Disease.** A) Scatter plot showing results of the 4 MR mendelian randomization methods, B) Forest plot showing individual single nucleotide polymorphism (SNP) ratio estimates (SNP-outcome effect estimate / SNP-exposure effect estimate), and C) Leave-one-out plot showing inverse variance weighted (IVW) estimates after omitting each SNP.


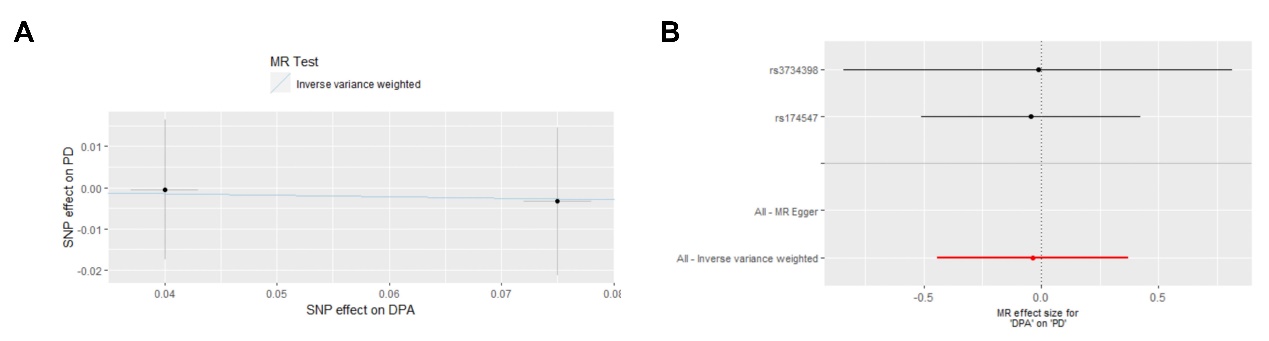


**Figure S5. Mendelian Randomization (MR) Sensitivity Plots of the Causal Effect of Docosapentaenoic Acid (DPA) Levels on Parkinson’s Disease.** A) Scatter plot showing results of the IVW method, and B) Forest plot showing individual single nucleotide polymorphism (SNP) ratio estimates (SNP-outcome effect estimate / SNP-exposure effect estimate).

# References

Guan, W., et al. (2014). Genome-wide association study of plasma N6 polyunsaturated fatty acids within the cohorts for heart and aging research in genomic epidemiology consortium. Circ Cardiovasc Genet. 7, 321-331. doi: 10.1161/CIRCGENETICS.113.000208.

Kettunen, J., et al. (2016). Genome-wide study for circulating metabolites identifies 62 loci and reveals novel systemic effects of LPA. Nat Commun. 7, 11122. doi: 10.1038/ncomms11122.

Lemaitre, R.N., et al. (2011). Genetic loci associated with plasma phospholipid n-3 fatty acids: a meta-analysis of genome-wide association studies from the CHARGE Consortium. PLoS Genet. 7, e1002193. doi: 10.1371/journal.pgen.1002193.

Nalls, M.A., et al. (2019). Identification of novel risk loci, causal insights, and heritable risk for Parkinson's disease: a meta-analysis of genome-wide association studies. Lancet Neurol. 18, 1091-1102. doi: 10.1016/s1474-4422(19)30320-5.
