# Supplementary material for: Associations between polyunsaturated fatty acid concentrations and Parkinson’s disease: A two-sample Mendelian randomization study
Source: Front Aging Neurosci. 2023 Feb 22;15:1123239. doi: 10.3389/fnagi.2023.1123239 (PMC9992541; doi:10.3389/fnagi.2023.1123239)
Supplement: Supplementary file 2 [file Data_Sheet_1.PDF]

STROBE-MR checklist of recommended items to address in reports of Mendelian randomization studies[1]

Item No.	Section		Checklist item	Page No.	Relevant text from manuscript
1	**TITLE and ABSTRACT**		Indicate Mendelian randomization (MR) as the study's design in the title and/or the abstract if that is a main purpose of the study	1	Abstract
	**INTRODUCTION**				
2	**Background**		Explain the scientific background and rationale for the reported study. What is the exposure? Is a potential causal relationship between exposure and outcome plausible? Justify why MR is a helpful method to address the study question	2	Parkinson's disease (PD) is one of the most common neurodegenerative diseases in the world. The effect of PUFAs on PD risk was controversial among studies.
3	**Objectives**		State specific objectives clearly, including pre-specified causal hypotheses (if any). State that MR is a method that, under specific assumptions, intends to estimate causal effects	2	We adopted this methodology to answer the question whether PUFAs were causally associated with PD risk.
	**METHODS**				
4	**Study design and data sources**		Present key elements of the study design early in the article. Consider including a table listing sources of data for all phases of the study. For each data source contributing to the analysis, describe the following:		
		a)	Setting: Describe the study design and the underlying population, if possible. Describe the setting, locations, and relevant dates, including periods of recruitment, exposure, follow-up, and data collection, when available.	S3-4 S9-10	Supplementary Table 2 and 4
		b)	Participants: Give the eligibility criteria, and the sources and methods of selection of participants. Report the sample size, and whether any power or sample size calculations were carried out prior to the main analysis	3	19 cohorts of three genome-wide association studies (GWAS) studies totaling 22,393 individuals
		c)	Describe measurement, quality control and selection of genetic variants	S3-4	Supplementary Table 2
		d)	For each exposure, outcome, and other relevant variables, describe methods of assessment and diagnostic criteria for diseases	S3-4 S9-10	Supplementary Table 2 and 4
		e)	Provide details of ethics committee approval and participant informed consent, if relevant	3	Ethical approvals were shown in the original publications.
5	**Assumptions**		Explicitly state the three core IV assumptions for the main analysis (relevance, independence and exclusion restriction) as well assumptions for any additional or sensitivity analysis	3	A valid MR analysis has three core assumptions.
6	**Statistical methods: main analysis**		Describe statistical methods and statistics used		

		a)	Describe how quantitative variables were handled in the analyses (i.e., scale, units, model)	S4	Levels of PUFAs were quantified as percentage of total fatty acids.
		b)	Describe how genetic variants were handled in the analyses and, if applicable, how their weights were selected	3-4	The exclusion criteria for SNPs
		c)	Describe the MR estimator (e.g. two-stage least squares, Wald ratio) and related statistics. Detail the included covariates and, in case of two-sample MR, whether the same covariate set was used for adjustment in the two samples	3-4	Mendelian Randomization Analyses
		d)	Explain how missing data were addressed	3-4	Proxy variants
		e)	If applicable, indicate how multiple testing was addressed	3-4	False discovery rate
7	**Assessment of assumptions**		Describe any methods or prior knowledge used to assess the assumptions or justify their validity	3-4	Mean F-statistics, I2GX, conditional F-statistics
8	**Sensitivity analyses and additional analyses**		Describe any sensitivity analyses or additional analyses performed (e.g. comparison of effect estimates from different approaches, independent replication, bias analytic techniques, validation of instruments, simulations)	3-4	MR-PRESSO, pleiotropy test, Cochrane's Q test and I2, scatter plots, forest plots, leave-one-out plots, funnel plots
9	**Software and pre-registration**				
		a)	Name statistical software and package(s), including version and settings used	5	Statistical Analyses
		b)	State whether the study protocol and details were pre-registered (as well as when and where)	NA	
	**RESULTS**				
10	**Descriptive data**				
		a)	Report the numbers of individuals at each stage of included studies and reasons for exclusion. Consider use of a flow diagram	5	Exposure summary-level data from 22,393 participants and outcome data from 33,674 cases and 449,056 controls
		b)	Report summary statistics for phenotypic exposure(s), outcome(s), and other relevant variables (e.g. means, SDs, proportions)	NA	
		c)	If the data sources include meta-analyses of previous studies, provide the assessments of heterogeneity across these studies	NA	
		d)	For two-sample MR:<br>i. Provide justification of the similarity of the genetic variant-exposure associations between the exposure and outcome samples	5	no participants overlap existed between exposure and outcome data

			ii. Provide information on the number of individuals who overlap between the exposure and outcome studies		
11	**Main results**				
		a)	Report the associations between genetic variant and exposure, and between genetic variant and outcome, preferably on an interpretable scale	S5-8	Supplementary Table 3
		b)	Report MR estimates of the relationship between exposure and outcome, and the measures of uncertainty from the MR analysis, on an interpretable scale, such as odds ratio or relative risk per SD difference	12	Table 2
		c)	If relevant, consider translating estimates of relative risk into absolute risk for a meaningful time period	NA	
		d)	Consider plots to visualize results (e.g. forest plot, scatterplot of associations between genetic variants and outcome versus between genetic variants and exposure)	S14-17	Scatter plots, forest plots, funnel plots
12	**Assessment of assumptions**				
		a)	Report the assessment of the validity of the assumptions	5-6	Mean F-statistics, I2GX, conditional F-statistics
		b)	Report any additional statistics (e.g., assessments of heterogeneity across genetic variants, such as I^2, Q statistic or E-value)	5-6	Cochrane's Q test and I2
13	**Sensitivity analyses and additional analyses**				
		a)	Report any sensitivity analyses to assess the robustness of the main results to violations of the assumptions	5-6	MR-PRESSO, pleiotropy test
		b)	Report results from other sensitivity analyses or additional analyses	5-6	Debiased IVW, MVMR
		c)	Report any assessment of direction of causal relationship (e.g., bidirectional MR)	NA	
		d)	When relevant, report and compare with estimates from non-MR analyses	7	Triangulation of the results with other researches
		e)	Consider additional plots to visualize results (e.g., leave-one-out analyses)	S14-17	Leave-one-out plots
	**DISCUSSION**				
14	**Key results**		Summarize key results with reference to study objectives	6-7	Our study utilized Mendelian randomization analyses to evaluate the causal relationship between omega-3 and omega-6 fatty acids and PD risk.
15	**Limitations**		Discuss limitations of the study, taking into account the validity of the IV assumptions, other sources of potential bias, and imprecision. Discuss both direction and magnitude of any potential bias and any efforts to address them	6-7	One limitation of our study

16	**Interpretation**				
		a)	Meaning: Give a cautious overall interpretation of results in the context of their limitations and in comparison with other studies	6-7	Triangulation of the results with other researches
		b)	Mechanism: Discuss underlying biological mechanisms that could drive a potential causal relationship between the investigated exposure and the outcome, and whether the gene-environment equivalence assumption is reasonable. Use causal language carefully, clarifying that IV estimates may provide causal effects only under certain assumptions	6-7	Several studies exploited the possible underlying mechanisms.
		c)	Clinical relevance: Discuss whether the results have clinical or public policy relevance, and to what extent they inform effect sizes of possible interventions	6-7	Our results could have implications for lifestyle intervention
17	**Generalizability**		Discuss the generalizability of the study results (a) to other populations, (b) across other exposure periods/timings, and (c) across other levels of exposure	6-7	The generalizability of the study results.
	**OTHER INFORMATION**				
18	**Funding**		Describe sources of funding and the role of funders in the present study and, if applicable, sources of funding for the databases and original study or studies on which the present study is based	7	Study funding
19	**Data and data sharing**		Provide the data used to perform all analyses or report where and how the data can be accessed, and reference these sources in the article. Provide the statistical code needed to reproduce the results in the article, or report whether the code is publicly accessible and if so, where	8	Data Availability
20	**Conflicts of Interest**		All authors should declare all potential conflicts of interest	7	Conflict of interest

This checklist is copyrighted by the Equator Network under the Creative Commons Attribution 3.0 Unported (CC BY 3.0) license.

1. Skrivankova VW, Richmond RC, Woolf BAR, et al. Strengthening the Reporting of Observational Studies in Epidemiology using Mendelian Randomisation (STROBE-MR): Explanation and Elaboration. BMJ. 2021;375:n2233.
